# AI Model Integrating Imaging and Clinical Data for Predicting CSF Diversion in Neonatal Hydrocephalus: A Preliminary Study

**DOI:** 10.1002/hbm.70363

**Published:** 2025-09-23

**Authors:** Yuwei Dai, Zhusi Zhong, Yan Qin, Yuli Wang, Guangdi Yu, Andrew Kobets, David W. Swenson, Jerrold L. Boxerman, Gang Li, Shenandoah Robinson, Harrison Bai, Li Yang, Weihua Liao, Zhicheng Jiao

**Affiliations:** ^1^ Department of Neurology Second Xiangya Hospital of Central South University Changsha Hunan China; ^2^ Russell H. Morgan Department of Radiology and Radiological Science Johns Hopkins University Baltimore Maryland USA; ^3^ Department of Diagnostic Imaging Brown University Health Providence Rhode Island USA; ^4^ Department of Radiology Xiangya Hospital of Central South University Changsha Hunan China; ^5^ National Clinical Research Center for Geriatric Disorders, Xiangya Hospital, Central South University Changsha Hunan China; ^6^ Department of Biomedical Engineering Johns Hopkins University Baltimore Maryland USA; ^7^ Department of Neurosurgery Montefiore Medical Center and the Albert Einstein College of Medicine New York USA; ^8^ Department of Radiology and Biomedical Research Imaging Center University of North Carolina at Chapel Hill Chapel Hill North Carolina USA; ^9^ Department of Neurosurgery Johns Hopkins University Baltimore Maryland USA; ^10^ National Engineering Research Center of Personalized Diagnostic and Therapeutic Technology, Xiangya Hospital of Central South University Changsha China

**Keywords:** artificial intelligence, cerebrospinal fluid shunting, MRI, neonatal hydrocephalus, raised intracranial pressure

## Abstract

Predictive tools for stratifying neonatal hydrocephalus into low‐ and high‐risk groups for cerebrospinal fluid (CSF) diversion are currently lacking. We developed and validated an artificial intelligence (AI) model that integrates multimodal imaging and clinical data to predict CSF diversion needs. The development cohort included 116 neonates with suspicion of raised intracranial pressure (ICP) from a Chinese tertiary referral hospital (80 with intracranial pressure > 80 mm H_2_O, 36 with intracranial pressure ≤ 80 mm H_2_O). The external validation cohort consisted of 21 neonates with hydrocephalus from an American medical center, categorized by etiology: prenatal myelomeningocele (MMC) closure (*n* = 5), postnatal MMC closure (*n* = 6), and post‐hemorrhagic hydrocephalus (PHH) (*n* = 10). Inclusion criteria required available MRI and complete clinical follow‐up to confirm CSF diversion outcomes. The primary outcome was the need for CSF diversion. Model performance was assessed using under the receiver operating characteristics curve (AUC), sensitivity, and specificity. The hybrid AI model achieved an AUC of 0.824 in the development cohort in predicting raised ICP, outperforming both the clinical‐only model (AUC 0.528, *p* < 0.001) and the image‐only model (AUC 0.685, *p* = 0.007). In the external validation cohort, the fused MRI‐based model achieved an AUC of 0.808. The model correctly predicted CSF diversion in 4/5 prenatal MMC, 4/6 postnatal MMC, and 9/10 PHH cases. The AI model demonstrated robust performance in predicting the need for CSF diversion, particularly in PHH cases, and has the potential to assist decision‐making, especially in settings with limited pediatric neurosurgical expertise. Future work should focus on further refining model performance for complex etiologies such as MMC‐associated hydrocephalus.

## Graphical abstract

Our study developed and validated an artificial intelligence (AI) model that integrates multimodal MRIs and clinical data to predict CSF diversion needs. The AI model was trained in a developmental cohort including 116 neonates with suspicion of raised intracranial pressure (ICP) and then validated in an external validation cohort consisting of 21 neonates with hydrocephalus.

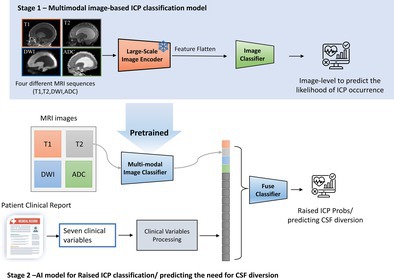

## 1 Introduction

Neonatal hydrocephalus is characterized by abnormal accumulation of cerebral spinal fluid (CSF), leading to progressive ventricular distension and typically associated with increased intracranial pressure (Kahle et al. 2024). An estimated 400,000 new cases of pediatric hydrocephalus occur annually worldwide, with over 80% arising in developing countries (Dewan et al. 2019). Infants with hydrocephalus are at high risk for neurodevelopmental delay, seizures, and cerebral palsy. Neonatal hydrocephalus is broadly classified into congenital and acquired forms, with myelomeningocele (MMC) commonly associated with congenital hydrocephalus and intraventricular hemorrhage typically leading to acquired hydrocephalus (Kahle et al. 2024). Management decisions, including CSF diversion through ventriculoperitoneal (VP) shunting or endoscopic third ventriculostomy (ETV), often rely on clinician expertise and institutional protocols. Delayed or unnecessary interventions increase the risk of shunt malfunction, infection, and other complications (Pindrik et al. 2022). There is a critical need for predictive tools to stratify infants into high‐risk groups requiring timely permanent CSF diversion and low‐risk groups suitable for primary care management; however, such tools are currently lacking.

Previous efforts have explored predictive models using clinical or radiological parameters. A generalized linear model (GLM) incorporating head circumference, corrected age, and weight achieved relatively accurate prediction of shunting success in infants with post‐hemorrhagic hydrocephalus (PHH) (Kayhanian et al. 2022). Similarly, changes in ventricular volume measured by neonatal ultrasound have been investigated to guide CSF diversion decisions (Roy et al. 2023). However, these models were developed in limited cohorts and have not been externally validated.

More recently, magnetic resonance imaging (MRI) has gained attention for evaluating shunt function and anatomy in pediatric hydrocephalus, offering detailed visualization over ultrasound (Pindrik et al. 2022; Wallace et al. 2014). Various MRI‐derived ventriculomegaly indices, including the frontal‐occipital horn ratio (FOHR) and the frontal‐temporal horn ratio (FTHR), serve as valuable adjunctive parameters in clinical decision‐making for shunt replacement procedures (Antes et al. 2012, 2013). However, manual quantification of these ventriculomegaly indices from MRI images is time‐consuming and subject to significant inter‐observer variability, limiting their feasibility in the clinical setting lacking pediatric neurosurgical expertise (Radhakrishnan et al. 2019). Furthermore, the necessity of sedation in neonates has impeded large‐scale MRI data collection, thereby constraining broader applications. In addition to these practical limitations, prior studies have predominantly focused on isolated etiological subgroups, without pursuing comprehensive analyses across diverse causes of neonatal hydrocephalus (Behjati et al. 2011; Roy et al. 2023).

Emerging advances in artificial intelligence (AI), particularly Vision‐Language Models (VLMs), offer new opportunities to overcome these challenges. By integrating imaging and clinical text data, VLMs enable more holistic analysis and exhibit superior few‐shot learning capabilities compared to traditional machine learning approaches (Bluethgen et al. 2024; Gupta et al. 2024). VLMs are particularly promising for supporting decision‐making in rare and heterogeneous conditions such as neonatal hydrocephalus (Rocamonde et al. 2023; Shakeri et al. 2024).

In this study, we developed and externally validated an AI model integrating multimodal clinical and imaging data to predict the need for permanent CSF diversion in neonates with hydrocephalus. Our goal was to create a tool that could assist clinicians in differentiating patients requiring specialized neurosurgical care from those suitable for local management.

## 2 Material and Methods

### 2.1 Development and Validation Cohort

Development cohort: A total of 116 neonates with suspected raised intracranial hypertension (ICP) secondary to potential intracranial infections from Xiangya Hospital of Central South University (XY) were included as the development cohort. Inclusion and exclusion criteria for this cohort have been previously described (Qin et al. 2023). Lumbar puncture was performed to measure ICP and collect CSF for cultures. Based on ICP measurements obtained via lumbar puncture, 80 neonates with ICP > 80 mm H_2_O were classified into the raised ICP group, while 36 neonates with ICP ≤ 80 mm H_2_O were classified into the non‐raised ICP group.

Validation cohort: A retrospective medical record review was conducted for all neonates born at Johns Hopkins University (JHU) between January 2018 and December 2020. Inclusion criteria were: (1) a clinical diagnosis of neonatal hydrocephalus (Pindrik et al. 2022); (2) at least one MRI examination; (3) regular and complete follow‐up documentation. A total of 21 neonates met these criteria and were included in the validation cohort. These neonates were further categorized into three subgroups: prenatal MMC closure, postnatal MMC closure, and PHH.

### 2.2 Standard Protocol Approvals, Registrations, and Patient Consents

This study was approved by institutional review boards (IRBs) of both participating hospitals. All procedures were conducted in accordance with the Declaration of Helsinki. Given the retrospective nature of the study, a waiver of informed consent was granted by the IRBs.

### 2.3 Data Processing

Prior to the development of an AI model for raised ICP classification and its subsequent validation for predicting the need for shunt surgery, both imaging data and clinical variables from the XY and JHU datasets were processed.

#### 2.3.1 Image Acquisition and Preprocessing

Four MRI sequences of interest were included: T1‐weighted, T2‐weighted, apparent diffusion coefficient (ADC), and diffusion‐weighted imaging (DWI). The details of MRI acquisition in two medical centers were shown in the Supporting Information. Given the variability in acquisition parameters across different MRI sequences and medical centers, image preprocessing was necessary to standardize spatial resolution, remove irrelevant regions, and extract meaningful information.

Anonymized DICOM files were processed sequentially based on spatial coordinates to reconstruct 3D MRI volumes. The preprocessing pipeline consisted of spatial rescaling, cropping, and intensity normalization. First, all MRI volumes were aligned to a standard axial plane and resampled to a uniform voxel spacing of 0.3 mm × 0.3 mm × 2 mm. To remove peripheral non‐relevant areas, the images were centrally cropped to retain a [512, 512, 64] voxel region centered on the rescaled MRI volume.

Substantial variability in intensity distributions was observed across MRIs due to differences in imaging protocols and scanner types. In addition, pixel intensities exhibited heavy‐tailed distributions, making traditional min‐max normalization unsuitable. To address this, pixel intensities above the 95th percentile were truncated for each image, and the truncated maximum value was then used to normalize pixel intensities to a [0, 1] range. This standardized preprocessing pipeline was consistently applied across all four imaging modalities to ensure data comparability and robustness in downstream analyses.

#### 2.3.2 Clinical Variables Processing

Seven relevant clinical variables were collected: gender, gestational age, head circumference, weight, mode of delivery, and Apgar scores at 1 and 5 min after birth. To ensure consistency, all variables were defined according to standardized criteria. Missing values in both datasets were imputed using the median values calculated from the corresponding variables in the internal dataset. To standardize the clinical variables, each feature was normalized by dividing its value by the maximum value observed in the internal dataset. This preprocessing step ensured that all clinical features were scaled to comparable ranges, facilitating their integration with imaging data for subsequent analyses.

### 2.4 Stage 1. Multimodal Image‐Based ICP Classification Model

**FIGURE 1:**
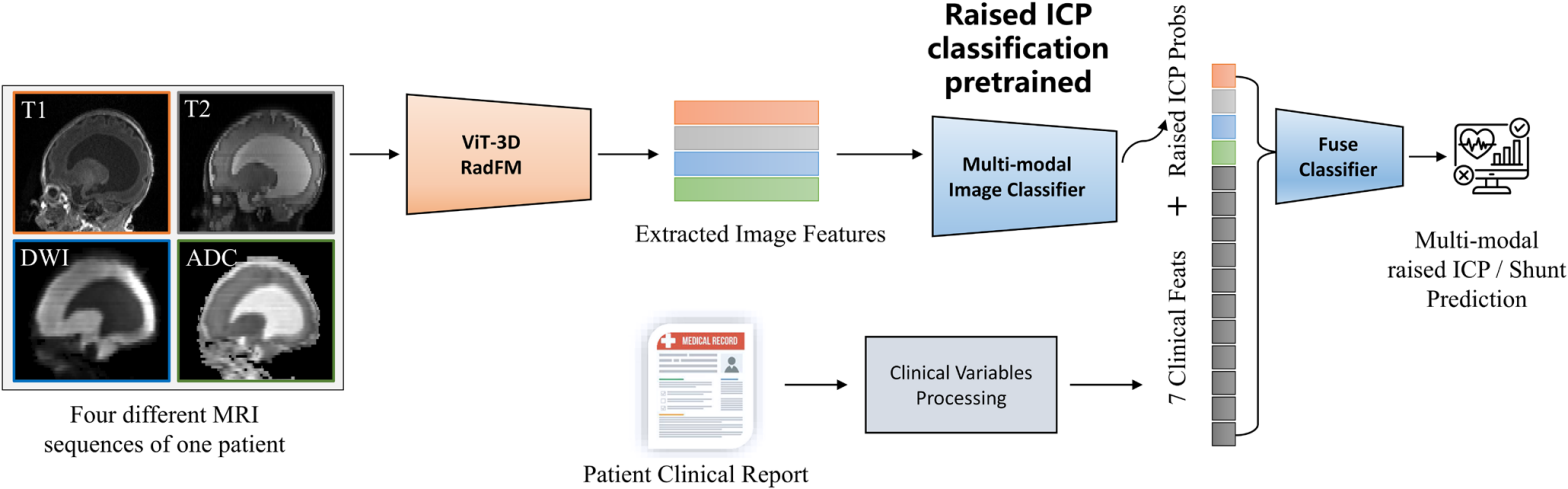
Pipeline figure.

Our multimodal ICP classification pipeline, shown in Figure 1, utilizes the Radiology Foundation Model (RadFM), a VLM pre‐trained on the large‐scale Medical Multi‐modal Dataset (MedMD) (Wu et al. 2023). The 3D Visual Transformer (ViT‐3D) encoder within RadFM extracts hierarchical spatial features from multimodal MRI data (Dosovitskiy 2020). An illustration of ViT‐3D has been shown in the Supporting Information.

ViT‐3D, based on a hierarchical attention mechanism, processes 3D volumetric inputs by capturing spatially contextualized features across multiple scales. Preprocessed MRI images were input into the ViT‐3D image encoder, generating 32 semantic feature vectors, each with a dimension of 5120. These features were then flattened and batch normalized. The image feature classifier module consists of a fully connected layer that reduced the feature dimension to 128, followed by batch normalization, ReLU activation, and dropout. A second fully connected layer further refined the 128‐dimensional features, again followed by ReLU activation, and a final linear layer mapped the features to output channels for probability prediction. The classifier ultimately generated a continuous probability score reflecting the likelihood of ICP occurrence.

### 2.5 Stage 2. Image–Clinical Hybrid Model for ICP Classification

Building upon the image‐based prediction model, we developed a hybrid multimodal model that integrated clinical variables to enhance the accuracy of raised ICP classification. In this hybrid framework, the image module first processed each MRI modality independently, generating modality‐specific raised ICP risk scores. The modality‐specific risk scores were then concatenated with seven normalized clinical features, which represented key indicators of the patient's systemic and neurological status, to form a combined feature vector. This fused feature vector was subsequently inputted into a fusion classifier designed to integrate multimodal information for the final raised ICP diagnosis. The fusion classifier consisted of a single hidden layer with 32 nodes, followed by batch normalization to promote training stability, a ReLU activation function, and a final linear output layer.

### 2.6 Raised ICP Classification Models Training

The goal of training the raised ICP classification models was to enable accurate regression of probability estimates using multimodal data. During model training, we used the cross‐entropy (CE) loss function, a standard choice for image classification tasks. The classification probability was estimated using the SoftMax function, and the CE loss was calculated between the predicted soft target and the ground‐truth label as follows:

$$L\left(y,z\right)=\sum_{i=0}^{M}-y_{i}\;\log\left(\frac{z_{i}}{\sum_{j}\exp\left(z_{j}\right)}\right)$$

where *M* represents the total class number of prediction classes. For instance, *M* = 2 when the classifier was trained to distinguish between ICP‐normal and ICP‐raised MR images. Here, $y_{i}$ denotes a one‐hot encoded vector representing the ground‐truth label, with the true class assigned a value of 1 and all others assigned 0. $z_{j}$ represents the logit, which is the raw output from the model's final layer for the *j*th class.

Model parameters were optimized using Adaptive Moment Estimation (Adam) with weight decay, a method that calculates adaptive learning rates for each parameter. The initial learning rate was set to 0.001, and the weight decay coefficient was also 0.001. Training was conducted over 100 epochs, and the model with the highest average Area Under the Receiver Operating Characteristic Curve (AUC) on the internal validation dataset was selected.

During training, the ViT‐3D image encoder was frozen to preserve the pre‐trained parameters obtained from RadFM on large‐scale cross‐modal tasks. For the image‐clinical hybrid model, the image classifier was initialized with parameters from the previously trained multi‐modal image model, thereby leveraging the learned feature representations to provide a strong initialization for the fusion‐based classification process.

### 2.7 External Validation: Predicting CSF Diversion

This AI model was externally validated on a cohort of 21 neonates from JHU. Patients were categorized into three subgroups based on etiology. The primary outcome was permanent CSF diversion. Subgroup analyses were performed to assess model performance within each etiological category. Imaging and clinical data from the validation cohort were processed using the same pipeline as the development cohort.

### 2.8 MRI‐Derived Ventriculomegaly Indices Measurement

Blinded assessment of MRI‐derived ventriculomegaly indices was performed by two neuroradiologists independently. For each neonate in the JHU cohort, mean values from both observers were utilized.

For the FOHR measurement on axial MRI images (Figure S1): maximal bifrontal ventricular width at the foramen of Monro (*a*), peak occipital horn diameters at the ventricular atrial level (*b*), and maximum biparietal cranial width (*c*). FOHR was calculated as: *FOHR =* (*a* + *b*)/(2*c). An MRI‐derived FOHR threshold of 0.55 was applied to guide permanent CSF diversion decisions in neonatal hydrocephalus (Wellons et al. 2017).

For the FTHR measurement on coronal MRI images (Figure S1): optimal frontal horn span at the Monro foramen level (*d*), maximal temporal horn breadth anterior to brainstem structures (*e*), and peak transverse biparietal measurement (*f*). FTHR was calculated: *FOHR =* (*d* + *e*)/(2*f).

### 2.9 Statistical Analysis

Categorical variables are presented as numbers and percentages, while continuous variables are reported as mean ± standard deviation. For intergroup comparisons, analysis of variance (ANOVA) analysis or Kruskal–Wallis test was used, depending on the normality of the data distribution. Fisher's exact test was applied for comparisons of categorical variables. Post hoc analyses were performed using either the Tukey Honest Significant Difference (HSD) test or the Dunn test, as appropriate, to further explore group differences. The predictive performance of the AI model was compared with FOHR using its established threshold of 0.55, and differences in paired accuracy were assessed using McNemar's test. All statistical analyses were conducted using R software (version 4.3.3), and a two‐sided *p* value of < 0.05 was considered statistically significant.

## 3 Results

### 3.1 Demographic Information and Clinical Characteristics

The demographic and clinical characteristics of the validation cohort are summarized in Table 1. Among the 21 neonates, 16 (76.2%) were female, with a median gestational age of 30.4 ± 5.6 weeks. The median head circumference and median birth weight were 29.0 ± 7.4 cm and 2.0 ± 1.3 kg. The median Apgar score at 1 min after birth was 5.1 ± 2.5, improving to 7.3 ± 1.7 at 5 min. Most neonates (66.7%; 14/21) were delivered via caesarean section, while the remaining 33.3% (7/21) underwent vaginal delivery. During a mean follow‐up of 5.2 ± 0.9 years, eight neonates (38.1%) required CSF shunting surgery.

**TABLE 1:** Demographic information and clinical characteristics of patients with neonatal hydrocephalus.

Variables	Total (*n* = 21)	Prenatal closure of MMC (*n* = 5)	Postnatal closure of MMC (*n* = 6)	Post‐hemorrhagic hydrocephalus (*n* = 10)	*p*
Gender					0.36
Male, *n* (%)	5 (23.8%)	0	2 (33.3%)	3 (30%)	
Female, *n* (%)	16 (76.2%)	5 (100%)	4 (66.7%)	7 (70%)	
Gestational age, mean ± SD, weeks	30.4 ± 5.6	31.3 ± 3.8	36.7 ± 3.3	26.2 ± 3.4	**< 0.001** *
Head circumference, mean ± SD, cm	29.0 ± 7.4	30.5 ± 2.8	35.4 ± 7.7	24.9 ± 5.7	**0.024** *
Birth weight, mean ± SD, kg	2.0 ± 1.3	1.7 ± 0.6	2.9 ± 0.9	1.5 ± 1.6	**0.03** *
Apgar_1 min, mean ± SD	5.1 ± 2.5	5.4 ± 2.2	7.0 ± 2.3	3.7 ± 2.2	**0.03** *
Apgar_5 min, mean ± SD	7.29 ± 1.7	7.2 ± 1.1	8.5 ± 0.8	6.6 ± 1.9	0.08
Model of delivery					0.60
Caesarean section	14 (66.7%)	4 (80%)	3 (50%)	7 (70%)	
Vaginal delivery	7 (33.3%)	1 (20%)	3 (50%)	3 (30%)	
CSF shunting	8 (38.1%)	1 (20%)	4 (66.7%)	3 (30%)	0.31
Interval between the first MRI and shunt surgery, mean ± SD, days	77.1 ± 117.7	135	4.5 ± 3.1	154.7 ± 164.9	**0.034** *
Interval between birth and last follow‐up, mean ± SD, years	5.2 ± 0.9	5.3 ± 0.9	4.6 ± 1.2	5.6 ± 0.6	0.13

*Note:* CSF: cerebral spinal fluid; IVH: intraventricular hemorrhage; MMC: myelomeningocele; MRI: magnetic resonance imaging.

*p < 0.05.

**FIGURE 2:**
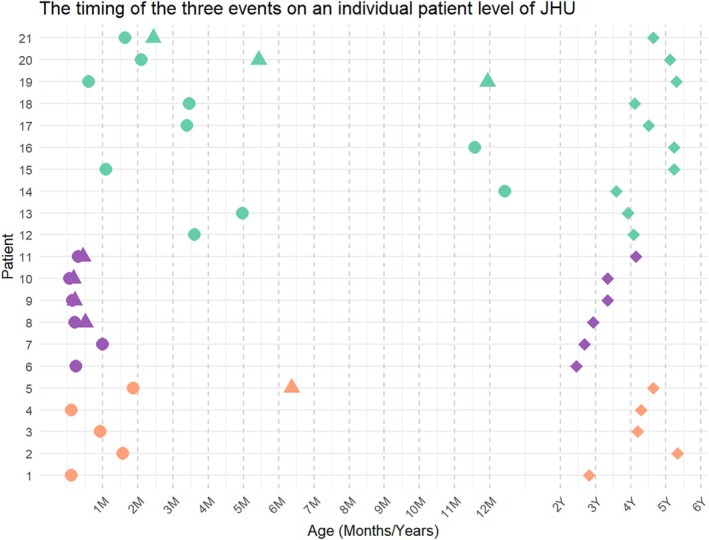
The timing of three major events (first MRI, shunt surgery, the last follow‐up) on an individual patient level of the validation cohort. Three subgroups are labeled with three distinct colors: Orange is for the prenatal myelomeningocele (MMC) closure subgroup; purple is for the postnatal MMC closure subgroup; green is for the post‐hemorrhagic hydrocephalus subgroup.

In the comparisons among three etiological subgroups, significant differences were observed in gestational age, weight at birth, head circumference, and Apgar score at 1 min (all *p* < 0.05). Post hoc analysis revealed that these differences were primarily driven by the postnatal MMC closure group compared to the PHH group. Notably, the interval between the first MRI and shunt surgery was significantly shorter in the postnatal MMC closure group compared to the PHH group (*p* = 0.034). Figure 2 illustrates the timing of three key events (first MRI, shunt surgery, last follow‐up) at the individual patient level.

### 3.2 Raised ICP Classification Models

**FIGURE 3:**
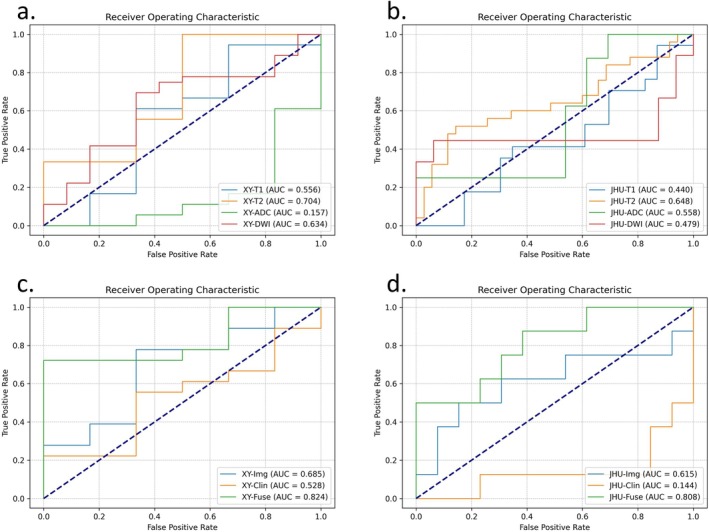
ROC curves. (a) ROC curves for single MRI modality in the developmental cohort; (b) ROC curves for single MRI modality in the validation cohort; (c) ROC curves of multi‐modal predictions in the developmental cohort; (d) ROC curves of multi‐modal predictions in the validation cohort.

In the development cohort of 116 neonates, the single‐modality model based on T2‐weighted images achieved the highest performance among individual modalities, with an AUC of 0.704, a sensitivity of 1.000, and a specificity of 0.500, indicating excellent sensitivity but moderate specificity (Table 2 and Figure 3a). The hybrid AI model, which integrated imaging and clinical data, achieved an AUC of 0.824 for predicting raised ICP, significantly outperforming both the clinical‐only model (AUC 0.528, *p* < 0.001) and the image‐only model (AUC 0.685, *p* = 0.007) (Table 3 and Figure 3c,d). The hybrid model demonstrated a sensitivity of 88% and a specificity of 84%, indicating strong performance for risk stratification in neonates with suspicion of raised ICP.

**TABLE 2:** The performance of image‐level models on ICP classification of the developmental cohort.

Modality	AUC (95% CI)	Accuracy (95% CI)	Sensitivity (95% CI)	Specificity (95% CI)	Precision (95% CI)	F1 score (95% CI)
T1	0.556 (0.242–0.874)	0.625 (0.167–0.958)	0.611 (0–1.000)	0.667 (0.167–1.000)	0.846 (0–1.000)	0.625 (0.167–0.958)
T2	0.704 (0.406–1.000)	0.875 (0.394–1.000)	1.000 (0.250–1.000)	0.5 (0.333–1.000)	0.857 (0.714–1.000)	0.875 (0.394–1.000)
ADC	0.157 (0–0.388)	0.208 (0.042–0.952)	0 (0–0.807)	0.833 (0.250–1.000)	0 (0–1.000)	0.208 (0.042–0.708)
DWI	0.634 (0.444–0.812)	0.688 (0.438–0.833)	0.694 (0.278–0.886)	0.667 (0.429–1.000)	0.862 (0.760–1.000)	0.688 (0.438–0.833)

**TABLE 3:** The performance of image‐based model, clinical‐based model and image‐clinical hybrid model on ICP classification of the developmental cohort.

Model	AUC (95% CI)	Accuracy (95% CI)	Sensitivity (95% CI)	Specificity (95% CI)	Precision (95% CI)	F1 score (95% CI)
Image‐based	0.685 (0.382–0.923)	0.750 (0.375–0.958)	0.778 (0.250–1.000)	0.667 (0.333–1.000)	0.875 (0.744–1.000)	0.750 (0.375–0.958)
Clinical‐based	0.528 (0.250–0.774)	0.583 (0.250–0.833)	0.556 (0.010–0.943)	0.667 (0.333–1.000)	0.833 (0.732–1.000)	0.583 (0.250–0.833)
Image‐clinical hybrid	0.824 (0.615–0.984)	0.792 (0.625–0.958)	0.880 (0.528–1.000)	0.840 (0.750–1.000)	1.000 (0.950–1.000)	0.792 (0.625–0.958)

### 3.3 CSF Diversion Prediction Models

In the external validation cohort of 21 neonates, the single modality based on T2‐weighted images also achieved the best AUC among single modalities (0.648) with an accuracy of 0.717, suggesting robust predictive performance of T2‐weighted images in the raised ICP prediction task as well as the CSF diversion prediction task (Table 4 and Figure 3b).

**TABLE 4:** The performance of image‐level prediction on the need of shunt surgery prediction of the validation cohort.

Modality	AUC (95% CI)	Accuracy (95% CI)	Sensitivity (95% CI)	Specificity (95% CI)	Precision (95% CI)	F1 score (95% CI)
T1	0.440 (0.253–0.627)	0.475 (0.400–0.700)	0.941 (0–1.000)	0.130 (0.083–0.952)	0.444 (0–0.786)	0.475 (0.400–0.700)
T2	0.648 (0.485–0.784)	0.717 (0.583–0.817)	0.520 (0.308–0.822)	0.857 (0.449–0.973)	0.722 (0.500–0.938)	0.717 (0.583–0.817)
ADC	0.558 (0.261–0.791)	0.571 (0.429–0.905)	1.000 (0.211–1.000)	0.308 (0.167–1.000)	0.471 (0.267–1.000)	0.571 (0.429–0.905)
DWI	0.479 (0.150–0.782)	0.760 (0.600–0.920)	0.444 (0.105–0.800)	0.938 (0.853–1.000)	0.800 (0.500–1.000)	0.760 (0.600–0.920)

The fused MRI‐based model achieved an AUC of 0.808 in the validation cohort, significantly outperforming the best‐performing single modality (AUC 0.615, *p* = 0.013). Integration of clinical data further enhanced sensitivity and specificity, underscoring the value of multimodal approaches for predicting permanent CSF diversion in neonates (Table 5).

**TABLE 5:** The performance of image‐based model, clinical‐based model and image‐clinical hybrid model on ICP classification of the validation cohort.

Model	AUC (95% CI)	Accuracy (95% CI)	Sensitivity (95% CI)	Specificity (95% CI)	Precision (95% CI)	F1 score (95% CI)
Image‐based	0.615 (0.324–0.887)	0.714 (0.524–0.905)	0.500 (0.133–1.000)	0.846 (0.406–1.000)	0.667 (0.296–1.000)	0.714 (0.524–0.905)
Clinical‐based	0.144 (0–0.360)	0.571 (0.333–0.762)	0 (0–0.478)	0.923 (0.667–0.943)	0 (0–0.750)	0.571 (0.333–0.762)
Image‐clinical hybrid	0.808 (0.586–0.976)	0.810 (0.619–0.952)	0.500 (0.375–1.000)	1.000 (0.400–1.000)	1.000 (0.400–1.000)	0.810 (0.619–0.952)

### 3.4 Predictive Performance Comparison: AI Model Versus FOHR

MRI‐derived FOHR correlated positively with FTHR (Figure S2). At the established FOHR threshold of 0.55, diagnostic accuracy reached 90.5%, with sensitivity of 85.7% and specificity of 80.8% (Table S1). No significant difference in predictive performance was observed between the AI model and FOHR (*p* = 0.324).

### 3.5 Subgroup Analysis and Prediction

**FIGURE 4:**
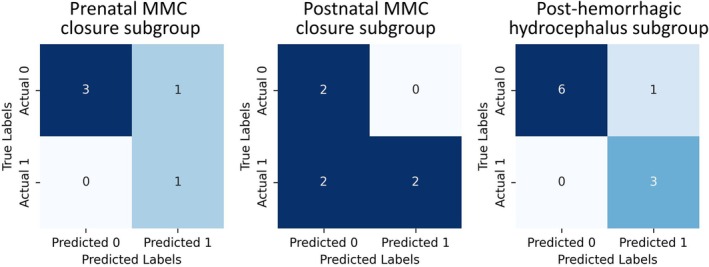
Confusion matrix of hybrid prediction for shunt surgery prediction on three subgroups of the validation cohort.

Figure 4 shows the confusion matrices for hybrid model prediction of shunt surgery across the three JHU subgroups: prenatal closure of MMC, postnatal closure of MMC, and PHH. The subgroup analysis revealed variability in model performance across different etiologies of neonatal hydrocephalus. In the prenatal MMC closure group (*n* = 5), only one neonate (20%) required shunting, with the model correctly predicting four out of five cases. Among postnatal MMC closure cases (*n* = 6), four neonates (66.7%) required shunting, and the model achieved correct predictions in four out of six cases. In the PHH group (*n* = 10), three neonates (30%) required CSF diversion, with the model accurately identifying 9 out of 10 cases.

### 3.6 Branch Analysis of the Image–Clinical Hybrid Model

**FIGURE 5:**
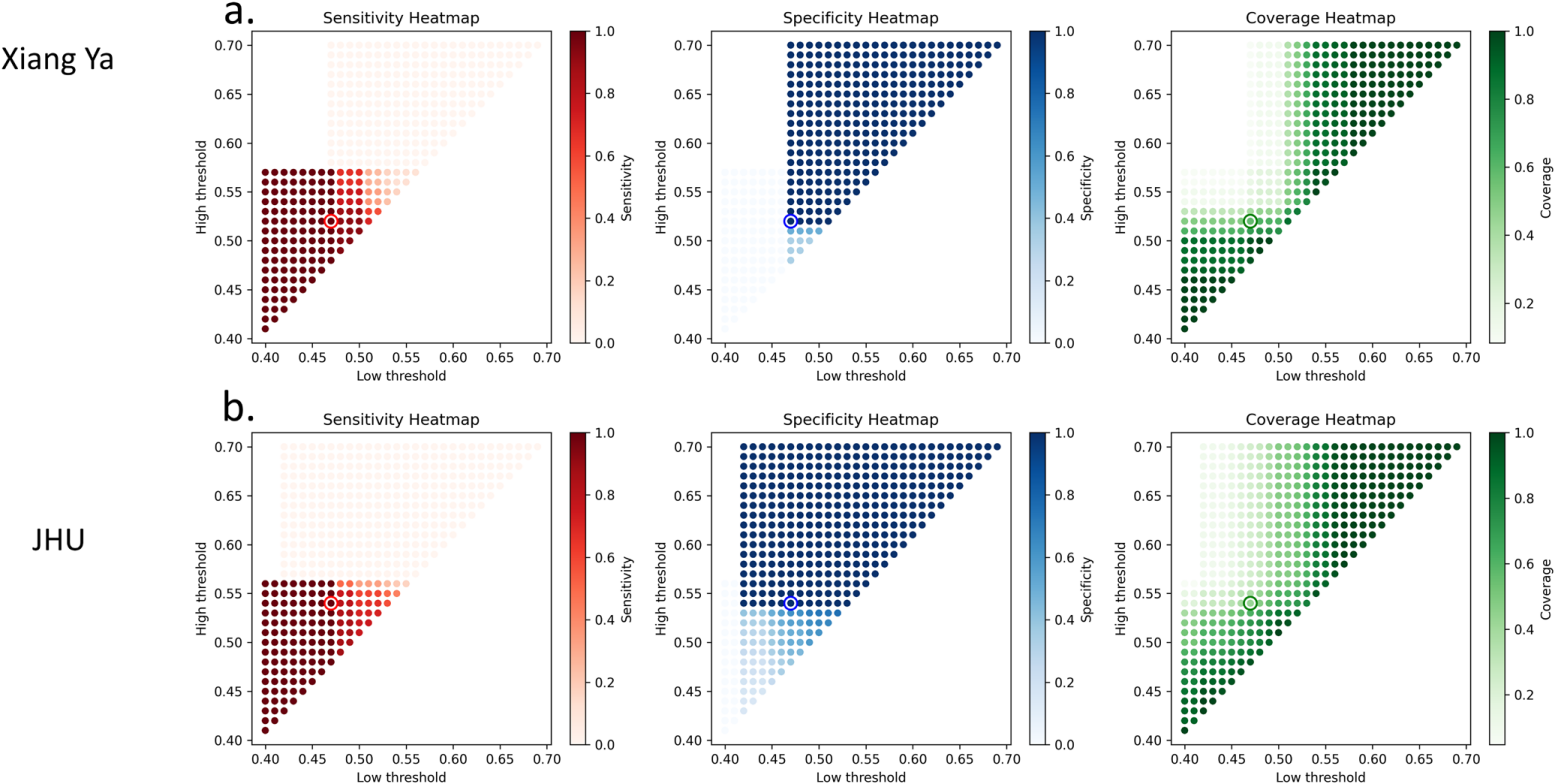
Branch analysis of the clinical–imaging hybrid model. Neonatal shunt stratification was stratified into high‐confidence need, high‐confidence no‐need, and intermediate groups. Heatmaps show the trade‐offs between sensitivity, specificity, and coverage when varying classification upper and lower thresholds: (a) developmental cohort, (b) validation cohort.

A branch analysis of the image–clinical hybrid model to stratify neonates into three categories was performed: high‐confidence need for shunt, high‐confidence no‐need for shunt, and intermediate. In the developmental cohort (Figure 5a), the optimal dual thresholds (low = 0.47, high = 0.52; marked by the circle) achieved 100% sensitivity, 100% specificity, 100% precision, and 58.3% coverage. In the external validation cohort (Figure 5b), the corresponding thresholds (low = 0.47, high = 0.54) also yielded 100% sensitivity, 100% specificity, and 100% precision, with 42.9% coverage. These heatmaps illustrate that stringent high thresholds preserved operating regions with perfect specificity, identifying neonates at high risk of requiring shunt, while low thresholds captured those confidently not requiring shunt. The intermediate group represents cases requiring closer monitoring.

## 4 Discussion

In this study, we developed an AI model originally trained to classify ICP based on clinical and radiological data. We successfully extrapolated its application to predict the need for CSF diversion in neonates with hydrocephalus. The model, utilizing the ViT‐3D image encoder of RadFM, captured imaging features critical for detecting elevated intracranial pressure. Given the strong pathophysiological relationship between raised ICP and the need for CSF diversion, the representations learned for raised ICP detection were transferable to this related clinical task. Validation in a cohort of 21 neonates from JHU demonstrated the feasibility of this approach, combining clinical and quantitative MRI features to enhance prediction accuracy. Notably, model performance varied across different etiologies of neonatal hydrocephalus, with the highest accuracy observed in the PHH subgroup. These findings suggest that AI models could play an important role in neonatal care, helping to identify high‐risk patients for early referral and appropriate intervention, ultimately reducing unnecessary transfers, alleviating family stress, optimizing resource utilization, and improving clinical outcomes.

Neonatal hydrocephalus presents with a range of symptoms and imaging findings related to raised ICP and ventriculomegaly. Broadly, pediatric hydrocephalus is classified into congenital and acquired forms with diverse etiologies (Kahle et al. 2024; Kim et al. 2018). In high‐income countries such as the United States, PHH following high‐grade intraventricular hemorrhage (IVH grade III/IV) remains a major contributor to neonatal hydrocephalus (Christian et al. 2016; Dewan et al. 2019). In our cohort, all infants with PHH also exhibited high‐grade IVH, reinforcing the strong correlation between severe IVH and subsequent hydrocephalus development (Kahle et al. 2024).

Subgroup comparisons revealed that variables such as head circumference, birth weight, and Apgar scores at 1 min were significantly different across etiologies, likely reflecting the lower gestational age of affected neonates. Interestingly, the interval between first MRI and shunt surgery was significantly longer in PHH cases compared to MMC‐associated hydrocephalus. This is consistent with current clinical practice, where CSF diversion in PHH is often delayed to allow neonates, typically preterm and of low birth weight (< 1500 g), to gain sufficient maturity, aiming to reduce shunt failure risk (Kayhanian et al. 2022; Lam and Heilman 2009; Pindrik et al. 2022).

Among neonates with MMC‐associated hydrocephalus, differences between prenatal and postnatal closure outcomes were observed. Only one of five infants (20%) with prenatal MMC closure required shunt surgery, compared to four of six infants (66.7%) with postnatal closure. Although not statistically significant, this trend aligns with previous reports suggesting that in utero repair of MMC may reduce the need for postnatal CSF diversion (Kahle et al. 2024). The optimal timing of shunt surgery following postnatal MMC repair remains controversial. While simultaneous shunt placement and MMC closure have been associated with favorable outcomes in some studies, a recent meta‐analysis found no significant differences in shunt failure or infection rates between simultaneous and delayed shunt placement (McCarthy et al. 2019).

CSF shunting remains the primary intervention for managing hydrocephalus, but invasive intracranial pressure monitoring, although informative, carries risks of infection and CSF leakage (Kayhanian et al. 2018). Non‐invasive imaging modalities such as cranial MRI, capable of evaluating CSF dynamics and anatomical changes, are gaining increasing attention for their predictive value (Patel et al. 2014; Yue et al. 2015). Recent studies have identified diffusion tensor imaging (DTI) as a useful predictor of shunt need in patients with prenatal MMC closure (Yuan et al. 2021). However, to date, no reliable model has been established using conventional MRI sequences and clinical variables to guide CSF diversion decisions, especially in settings with limited pediatric neurosurgical expertise.

Our results demonstrate that hybrid AI models integrating imaging and clinical data significantly outperform image‐only or clinical‐only models, particularly in terms of sensitivity. This performance gain likely stems from the complementary nature of these data sources: MRI captures detailed anatomical and physiological changes, while clinical data provides context regarding patient status, symptomatology, and broader medical history. By synthesizing both modalities, the hybrid model can achieve more accurate and informative predictions for complex clinical decision‐making.

To evaluate the potential clinical applicability of our hybrid AI model, we compared its performance with MRI‐derived FOHR. The hybrid AI model demonstrated comparable overall performance with no significant differences (*p* > 0.05), although FOHR showed relatively higher sensitivity. The higher specificity maintained by our AI model suggests more stringent selection criteria for VP shunt candidates. We hypothesize that incorporating clinical variables may shift the model's predictive tendency toward more conservative surgical criteria. In neonatal hydrocephalus management, this characteristic may be clinically advantageous by reducing unnecessary surgical interventions and associated shunt complications (Hari‐Raj et al. 2021; McCarthy et al. 2019). Furthermore, considering conventional radiological indices requiring manual measurement and specialized pediatric neurosurgical expertise, the comparable performance of our AI model underscores its potential for improved reproducibility in clinical practice.

In the PHH subgroup, the hybrid model achieved high sensitivity and specificity, likely attributable to the relatively uniform imaging findings—ventricular dilation and blood product deposition—and consistent clinical presentation associated with PHH (Christian et al. 2016; Kahle et al. 2024). In contrast, predictive performance in the MMC subgroups was lower, reflecting several challenges: small sample sizes, imbalanced outcome distributions, and the short time interval between imaging and surgical intervention, which limited the model's ability to detect early predictors of CSF diversion. These findings underscore the need for larger datasets and further model refinement, particularly for heterogeneous clinical subgroups. We also admitted that the lower shunt rate in the prenatal MMC closure group may lead to model underprediction.

This study has several limitations. First, LP was used as the ground truth for intracranial hypertension in the training cohort, despite being invasive, infrequent in practice, and potentially inconsistent with clinical decision‐making. Second, the small and unbalanced external validation cohort, particularly in the MMC subgroups, raises concerns about overfitting and limits generalizability. Third, key clinical predictors such as hemorrhage grade, infection, and structural anomalies were not included, which may have reduced model performance. Fourth, the model's interpretability could be improved through the analysis of its performance across a broader spectrum of MRI‐derived ventriculomegaly parameters. Additionally, training relied on a single imaging modality (MRI) and single‐center data, restricting generalizability, especially where ultrasound or CT are preferred. Selection bias is also a concern, as including only infants who underwent MRI likely excluded lower acuity cases evaluated by ultrasound, skewing the cohort toward more severe presentations.

## 5 Conclusion

In conclusion, we developed and externally validated a multimodal AI model for predicting the need for CSF diversion in neonatal hydrocephalus. By integrating imaging and clinical data, the model showed encouraging performance for risk stratification, particularly in the PHH subgroup. While the results suggest potential for supporting clinical decision‐making, further validation in larger, more diverse cohorts is needed before broader clinical application. Future work should focus on adapting the model for use in resource‐limited settings, such as by incorporating head ultrasound, and on improving performance in more complex clinical subgroups to enhance its generalizability and utility.

## Author Contributions

All authors fulfill the authorship criteria. Y.D., Z.Z., Y.Q.: writing original draft, acquisition of data, analysis or interpretation of data. W.L. and L.Y.: study concept and design, revising the manuscript. Y.W., G.Y., A.K.: analysis or interpretation of data, preparing the figures/tables. D.W.S., J.B., G.L., S.R., L.Y., W.L., H.B., and Z.J.: revising the manuscript.

## Ethics Statement

This study has been approved by institutional review boards (IRBs) of Xiangya Hospital and JHU. All procedures were conducted according to the guidelines of the Declaration of Helsinki and consents from all patients' guardians were obtained.

## Conflicts of Interest

The authors declare no conflicts of interest.

## Supporting information


**Data S1:** hbm70363‐sup‐0001‐Supinfo.docx.

## Data Availability

The data that support the findings of this study are available on request from the corresponding author. The data are not publicly available due to privacy or ethical restrictions.
