# Supplementary material for: AI Model Integrating Imaging and Clinical Data for Predicting CSF Diversion in Neonatal Hydrocephalus: A Preliminary Study
Source: Hum Brain Mapp. 2025 Sep 23;46(14):e70363. doi: 10.1002/hbm.70363 (PMC12455681; doi:10.1002/hbm.70363)
Supplement: Supplementary file 1 — Data S1: hbm70363‐sup‐0001‐Supinfo.docx. [file HBM-46-e70363-s001.docx]

**Supplementary materials**

1. **The details of MRI acquisition at Xiangya Hospital of Central South University (XY)**

MRI data was acquired with a Siemens Prisma 3.0-T scanner (Siemens Health

care, Erlangen, Germany) using a 64-channel head/neck receiver coil. The data for each subject consisted of a sagittal, three-dimensional, T1-weighted magnetization-prepared 2 inversion-contrast rapid gradient-echo (T1-weighted MP2RAGE) with the following parameters: TR/TE=3600ms/3.15ms, flip angle = 4, TI =1100ms/300

ms, BW=240Hz, voxel size=0.78×0.78×0.78, slice thickness=0.78mm. Axial T2-weighted images were acquired using a turbo spin echo (TSE) sequence with following parameters: TR=4000ms, TE=93ms, flip angle =150, pixel spacing=0.42 × 0.42 mm, voxel size = 0.7 × 0.7 × 2, slice thickness = 4 mm. Diffusion-weighted images (DWI) were acquired with following parameters: TR/TE = 3850/51ms, b-values of 0 and 1000 s/mm², slice thickness = 4 mm, and in-plane resolution = 1.1 × 1.1 mm. Apparent diffusion coefficient (ADC) maps were derived from diffusion-weighted images acquired using a RESOLVE sequence (b = 0 and 1000 s/mm²; TR/TE = 3850/51ms; in-plane resolution = 1.1 × 1.1 mm; slice thickness = 4 mm; no interslice gap).

1. **The details of MRI acquisition at Johns Hopkins University (JHU)**

MRI images were acquired with a Siemens 1.5-T scanner. All neonates underwent T1-weighted, T2-weighted, DWI, and ADC sequences. T1-weighted images were collected with these parameters: TR/TE = 500/13ms, flip angle = 137, bandwidth = 240 Hz, voxel size = 0.78 × 0.78 × 0.78 mm, slice thickness=2.5mm. T2-weighted images: TR=2800ms, TE=92ms, flip angle =150, pixel spacing=0.78 × 0.78mm, voxel size = 0.7 × 0.7 × 2, slice thickness = 2 mm. DWI was performed using a single-shot echo-planar RESOLVE sequence with b-values of 0 and 1000 s/mm² ,TR/TE = 9100/86ms, slice thickness = 2 mm, and in-plane resolution = 1.1 × 1.1 mm. Apparent diffusion coefficient (ADC) maps were derived from axial diffusion-weighted images acquired using a RESOLVE sequence (b-values = 0 and 1000 s/mm²; TR/TE = 9100/86ms; in-plane resolution = 1.1 × 1.1 mm; slice thickness = 2 mm; no interslice gap).

1. **ViT-3D in the Radiology Foundation Model (RadFM): Architecture and Training Methodology**

The ViT-3D model, central to the Radiology Foundation Model (RadFM), is an advanced extension of the Vision Transformer (ViT) architecture tailored for volumetric medical imaging tasks. Unlike conventional 2D ViTs, ViT-3D processes three-dimensional image volumes (e.g., CT or MRI) by segmenting them into fixed-size non-overlapping 3D patches (e.g., 16×16×16 voxels). Each 3D patch is flattened and projected into a latent embedding space via a learnable linear projection layer. These patch embeddings are supplemented with learnable 3D positional encodings that capture spatial relationships across all three axes, preserving anatomical coherence.

The core architecture is built upon a Transformer encoder consisting of six identical layers, each comprising a multi-head self-attention mechanism followed by a feed-forward multilayer perceptron (MLP), with residual connections and layer normalization applied after each sub-block. The use of multiple attention heads enables the model to simultaneously capture information from various representation subspaces. For visual encoding, we employ a 12-layer ViT module with 768 feature dimensions. To aggregate the visual features, we adopt a 6-layer Perceiver transformer decoder that uses a learnable latent array of shape 32 × 5120. Consequently, each 3D image is ultimately embedded into a fixed-size feature representation of 32 × 5120 after passing through the visual encoder and Perceiver-based aggregation.

During pretraining, RadFM employs a masked autoencoding strategy adapted for 3D data. A high percentage (e.g., 75–90%) of the 3D patches are randomly masked, and only the unmasked patches are passed through the encoder. A lightweight decoder—composed of fewer Transformer layers—is then used to reconstruct the full volume from the encoded visible patches and learned mask tokens. The reconstruction objective, typically Mean Squared Error (MSE) or L1 loss, encourages the model to learn semantically meaningful representations of anatomical and pathological structures without requiring manual labels.

The pretrained ViT-3D encoder can be fine-tuned for various downstream radiological tasks, such as lesion classification, multi-organ segmentation, report generation, or survival prediction. This self-supervised pretraining paradigm enables the model to generalize effectively across institutions, imaging protocols, and patient populations, providing a scalable foundation for clinical AI applications in radiology.

| **MRI-derived index** | **Threshold** | **Accuracy**  **(95% CI)** | **Sensitivity**  **(95% CI)** | **Specificity**  **(95% CI)** | **Precision**  **(95% CI)** | **F1 score**  **(95% CI)** |
| --- | --- | --- | --- | --- | --- | --- |
| frontal-occipital horn ratio (FOHR) | 0.550 | 0.905 (0.762–1.000) | 0.857 (0.500–1.000) | 0.808 (0.653–1.000) | 0.857 (0.600–1.000) | 0.857 (0.500–1.000) |

**Supplementary table 1. The predictive performance of the frontal-occipital horn ration to predict the need for permanent CSF shunting in validation cohort.**

**Supplementary Figure S1. The frontal-occipital horn ratio (FOHR) and the frontal-temporal horn ratio (FTHR) measurement. (a) FOHR measurement; (b) FTHR measurement.**


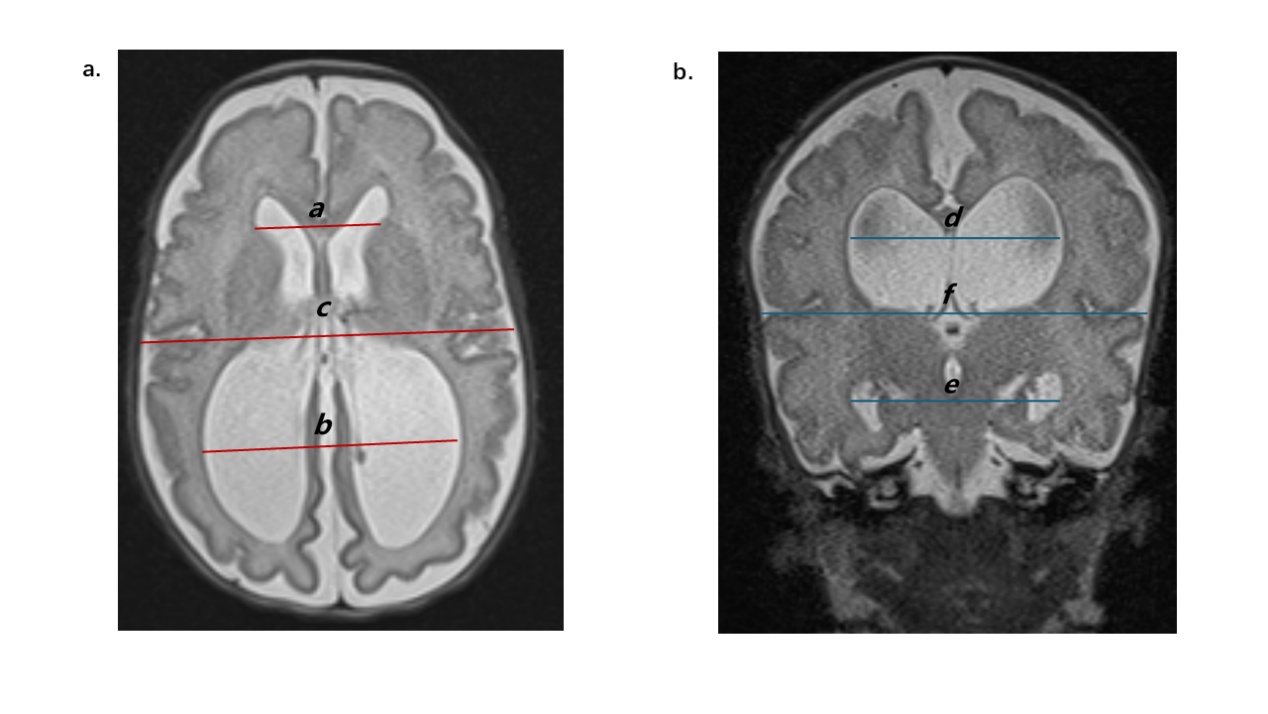


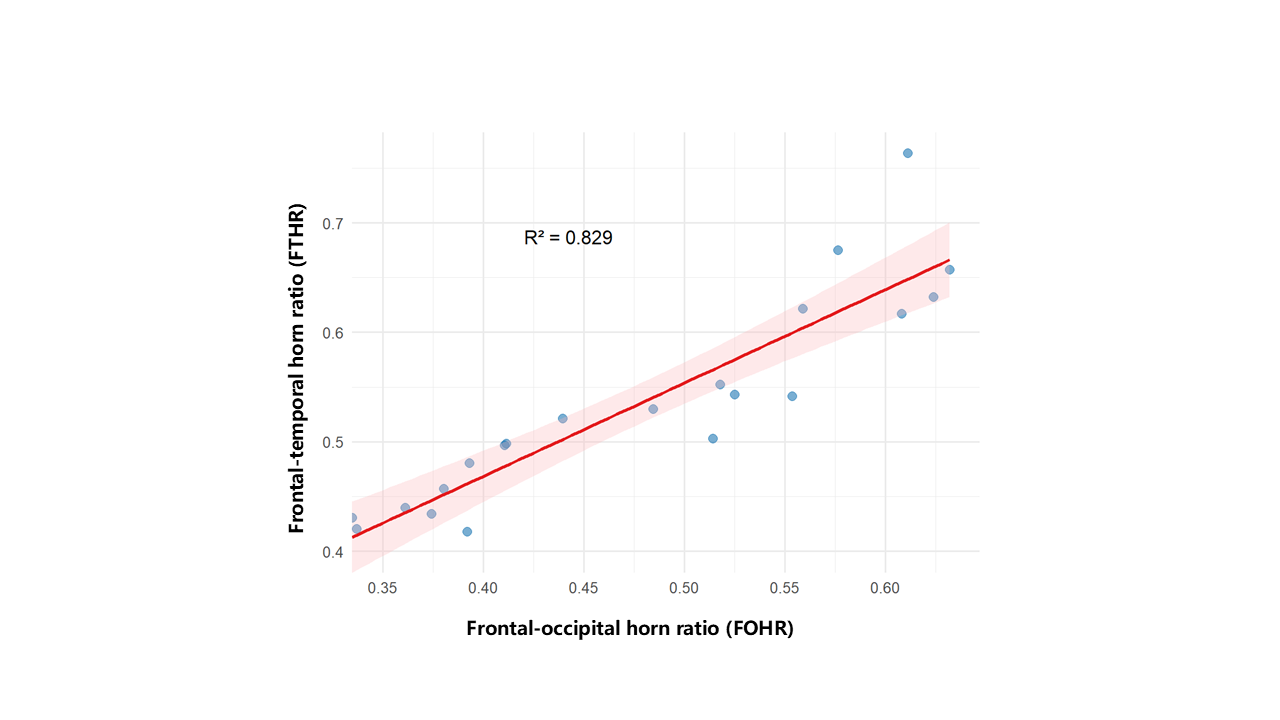
**Supplementary Figure S2. Correlation plots of MRI-derived frontal occipital horn ratios (FOHRs) with frontal temporal horn ratios (FTHRs).**
